# Longitudinal, population-based study of racial/ethnic differences in colorectal cancer survival: impact of neighborhood socioeconomic status, treatment and comorbidity

**DOI:** 10.1186/1471-2407-7-193

**Published:** 2007-10-16

**Authors:** Scarlett Lin Gomez, Cynthia D O'Malley, Antoinette Stroup, Sarah J Shema, William A Satariano

**Affiliations:** 1 Northern California Cancer Center, Fremont, California, USA; 2 Health Research and Policy, Stanford University School of Medicine, Stanford, California, USA; 3 Utah Cancer Registry, University of Utah, Salt Lake City, Utah, USA; 4 Division of Epidemiology, School of Public Health, University of California, Berkeley, California, USA

## Abstract

**Background:**

Colorectal cancer, if detected early, has greater than 90% 5-year survival. However, survival has been shown to vary across racial/ethnic groups in the United States, despite the availability of early detection methods.

**Methods:**

This study evaluated the joint effects of sociodemographic factors, tumor characteristics, census-based socioeconomic status (SES), treatment, and comorbidities on survival after colorectal cancer among and within racial/ethnic groups, using the SEER-Medicare database for patients diagnosed in 1992–1996, and followed through 1999.

**Results:**

Unadjusted colorectal cancer-specific mortality rates were higher among Blacks and Hispanic males than whites (relative rates (95% confidence intervals) = 1.34 (1.26–1.42) and 1.16 (1.04–1.29), respectively), and lower among Japanese (0.78 (0.70–0.88)). These patterns were evident for all-cause mortality, although the magnitude of the disparity was larger for colorectal cancer mortality. Adjustment for stage accounted for the higher rate among Hispanic males and most of the lower rate among Japanese. Among Blacks, stage and SES accounted for about half of the higher rate relative to Whites, and within stage III colon and stages II/III rectal cancer, SES completely accounted for the small differentials in survival between Blacks and Whites. Comorbidity did not appear to explain the Black-White differentials in colorectal-specific nor all-cause mortality, beyond stage, and treatment (surgery, radiation, chemotherapy) explained a very small proportion of the Black-White difference. The fully-adjusted relative mortality rates comparing Blacks to Whites was 1.14 (1.09–1.20) for all-cause mortality and 1.21 (1.14–1.29) for colorectal cancer specific mortality. The sociodemographic, tumor, and treatment characteristics also had different impacts on mortality within racial/ethnic groups.

**Conclusion:**

In this comprehensive analysis, race/ethnic-specific models revealed differential effects of covariates on survival after colorectal cancer within each group, suggesting that different strategies may be necessary to improve survival in each group. Among Blacks, half of the differential in survival after colorectal cancer was primarily attributable to stage and SES, but differences in survival between Blacks and Whites remain unexplained with the data available in this comprehensive, population-based, analysis.

## Background

Cancers of the colon and rectum are the second leading cause of cancer deaths among men and women in the United States (US), responsible for more than 57,000 deaths each year [1,2]. Despite declines in the incidence and mortality of colorectal cancer, survival following diagnosis has improved only modestly over the past 15 years [1-4]. However, early detection measures are available for colorectal cancer, and if caught in the earliest stage, more than 90% of patients will survive beyond five years.

Decreased survival is well documented among Blacks in relation to Whites, with most recent population-based studies showing a 30–50% higher rate of disease-specific mortality after diagnosis [5-13], and the largest racial/ethnic differentials seen among stage I and II cancers [5]. Of particular concern, the disparity in mortality rates between Blacks and Whites have widened over time [14,15]. The reasons for these differences are not well understood. The limited number of studies that have examined access and quality of cancer treatment, such as socioeconomic status [9-13,16], general treatment modalities [10], health insurance [13] and provider characteristics [9] have reported inconsistent results as to the extent to which these factors explained the Black-White differences in survival. Although comorbid illness and tumor aggressiveness have been shown to independently affect survival, their impact on racial/ethnic survival differences have not been well addressed.

There have been fewer population-based studies of colorectal survival in other racial/ethnic groups [5,6,17-20]. In a recent analysis of SEER data examining differences survival across detailed racial/ethnic groups, Chien et al found that, in addition to Blacks, higher hazard ratios for stage-adjusted colorectal cancer-specific deaths were seen among American Indians, Hawaiians, and Mexicans [5]. The authors speculated that the remaining decreased survival in these groups may be due to socioeconomic status and/or comorbidities. Choe et al found, in an analysis based on imputed SEER birthplace data, that foreign-born Asians were slightly more likely than US-born to present with advanced stage and have decreased stage-adjusted survival [17]. As colorectal cancer occurrence is known to be related to factors associated with living in a more developed country (sedentary lifestyle, high consumption of meats and saturated fats, and low consumption of fruits and vegetables [21,22]), disease burden in Hispanic and Asian racial/ethnic groups is expected to increase as they become acculturated to the lifestyle more common in the US [22-24]. It is important to also emphasize that "Asian" and "Hispanic" are very heterogeneous categories, each consisting of a variety of separate and discrete racial/ethnic groups. Thus, the understanding of issues related to colorectal cancer is a priority for these populations. Currently, in SEER data, although detailed Hispanic origin is collected, more than half of Hispanic patients are coded as "other or not otherwise specified" for detailed Hispanic origin, thus analyses for detailed Hispanic subgroups is not advisable. However, detailed Asian categories are collected and are mostly accurate and reliable [25], thus analyses for detailed Asian subgroups is possible.

Our study aimed to address the gaps in understanding of factors associated with racial/ethnic survival patterns by jointly considering patient and clinical factors including sociodemographics, tumor characteristics, neighborhood SES, treatment, and comorbidities, in a representative and large population of Medicare-eligible patients (aged 65 and older, who comprise about 70% of all colorectal patients [26]). Using the SEER-Medicare linked database, our analyses extend those of Chien et al by including data on area-based socioeconomic status and comorbidities. We also considered the effects of these factors on survival within each of six racial/ethnic groups [27].

## Methods

### Data Source

We used the linked Surveillance, Epidemiology, and End Results (SEER) – Medicare database, which combines data from the NCI SEER Program of population-based registries that collect demographic, clinical, and vital status information for persons with cancer, with Medicare claims for covered health care services, including hospitalizations [28-30]. The registries in the SEER Program, at the time of this analysis, included the states of Connecticut, Hawaii, Iowa, New Mexico and Utah, and the metropolitan areas of Atlanta, Detroit, San Francisco/Oakland, Seattle/Puget Sound, San Jose/Monterey, and Los Angeles, representing approximately 14% of the US population [26]. The latter two registries joined the SEER program in 1992. Medicare is the primary source of health insurance for 97% of Americans age 65 years and older. All recipients are enrolled in Part A benefits, which cover inpatient care in hospitals and other facilities; ninety-five percent of beneficiaries also subscribe to Part B, which covers additional services including physician services [29]. These are the sources of admissions data used in this analysis.

The linkage of SEER with Medicare data, which is based on a deterministic matching algorithm using social security number, name, sex, and date of birth [28], captured 93% of patients age 65 and older at diagnosis in the SEER database [29]. This analysis used data from the linkage conducted in 1999. Recipients under age 65 who received Medicare benefits for end-stage renal disease or for other reasons were excluded.

### Study Subjects

Patients living in a SEER catchment area and aged 65 and older when diagnosed with a microscopically-confirmed, first primary, malignant tumor of the colon or rectum (ICD-O-2 codes C180–C209, C260, excluding histologies 9590–9989) during the period January 1, 1992 through December 31, 1996 were included. This period of time was included for consistency with availability of physician claims data and 1990 Census data, which are provided as part of the linked database. We further excluded about 10% of cases that were coded as lymphomas, sarcomas, melanomas, carcinoids, or tumors of the squamous cell, appendix, or unspecified sites; those with invalid survival time (including those diagnosed at death or autopsy); those with unknown cause of death; and those of unknown or other race/ethnicity (N = 208), leaving us a final sample of 41,901 subjects. The numbers of patients in each racial/ethnic group are shown in Table 1.

**Table 1:** Percent distribution of colorectal cancer patients, demographic and clinical characteristics by race/ethnicity (N = 41,901), SEER, 1992–1996

**Characteristic**	**Non-Hispanic White **	**Non-Hispanic Black **	**Hispanic^1 ^**	**Chinese **	**Japanese **	**Filipino **
	**N = 34,164**	**N = 3,184**	** N = 2,061**	**N = 881**	**N = 1,116**	**N = 495**
Gender						
Male	47	43	50	57	54	56
Female	53	57	50	43	46	44

Year of diagnosis						
1992–93	41	40	38	38	40	36
1994–96	59	60	62	62	60	64

SEER region^2^						
SFBA	15	14	22	50	10	35
Connecticut	16	6	5	0	0	1
Detroit	13	32	1	1	0	0
Hawaii	1	0	0	13	60	33
Iowa	16	2	1	--	0	--
New Mexico	3	1	18	0	0	--
Seattle	11	3	1	4	5	4
Utah	3	0	2	0	1	--
Atlanta	4	13	1	1	--	0
LA	18	29	49	31	24	27

20%+ below poverty (N = 38,964)	7	52	25	20	9	11

Below poverty quartiles^3 ^(N = 38,964)						
1 (highest SES)	28	5	11	16	30	18
2	27	7	16	22	28	29
3	26	14	23	23	21	28
4 (lowest SES)	19	74	50	38	21	25

Non-HS quartiles^4 ^(N = 38,964)						
1 (highest SES)	28	6	11	20	19	11
2	27	11	17	20	22	21
3	26	15	19	21	28	22
4 (lowest SES)	18	69	53	39	31	47

Median income quartiles^5 ^(N = 38,964)						
1 (highest SES)	27	6	14	29	36	30
2	26	11	23	26	32	37
3	25	21	30	20	20	17
4 (lowest SES)	22	62	33	26	12	16

SES index^6 ^quartiles (N = 38,964)						
1 (highest SES)	34	7	15	29	34	21
2	21	7	13	13	20	24
3	29	19	27	27	31	34
4 (lowest SES)	16	67	45	32	15	22

Unknown SES	7	10	12	17	13	20

Married at diagnosis						
Yes	52	37	51	66	64	64
No	45	59	46	32	35	34
Unknown	2	4	3	2	1	1

Age at diagnosis (mean ± S.D.)						
65–69 (67.1 ± 1.4)	20	26	30	19	27	27
70–74 (72.0 ± 1.4)	24	26	25	25	29	22
75–79 (77.0 ± 1.4)	23	22	19	26	20	19
80+ (84.9 ± 4.1)	33	27	26	31	23	32

Site (location)						
Proximal colon	46	49	42	36	36	27
Distal colon	28	30	27	35	35	38
Rectum	26	21	31	29	29	34

AJCC stage at diagnosis						
Stage I	24	21	22	21	27	23
Stage II	32	28	31	31	29	25
Stage III	22	22	24	27	26	28
Stage IV	15	20	17	13	12	16
Unknown	7	9	7	8	6	7

Grade/differentiation						
Well	10	10	9	7	6	6
Moderate	60	63	61	65	72	68
Poor/None	19	14	19	19	13	15
Unknown	12	13	11	9	9	11

Surgery^7^						
None/NC Direct	9	14	9	10	6	11
Partial	7	6	6	6	8	7
GT Part/LT Tot	72	69	70	74	75	71
Total	11	11	14	10	11	10
Surgery, NOS	0.2	0.2	0.3	0.2	0.1	0.4

Radiation^8^						
None	90	91	86	90	91	89
Any	9	7	11	8	8	9
Unknown	1	1	2	1	1	2

Chemotherapy^9^						
None	92	96	87	89	95	92
Any	8	4	13	11	5	8

Received guideline treatment, stage III colon cancer (N = 6940)^10^	16	10	23	21	10	15

Received guideline treatment, stages II and III rectal cancer (N = 4626) ^10^	8	3	10	7	4	4

Received guideline treatment, stage III colon + stages II/III rectal cancer (N = 11,566) ^10^	13	8	17	15	8	10

Comorbidity^11^						
None	68	68	72	82	77	80
1	22	19	19	14	16	13
2 or more	10	13	9	5	5	7

All of the comparisons are statistically significant across races/ethnicities (Chi-Square p < 0.001).

^1 ^Hispanic = 2053 Whites, 8 Blacks

^2 ^SFBA: San Francisco Bay Area (San Francisco-Oakland & San Jose-Monterey regions), LA: Los Angeles region

^3 ^Percent of census tract residents living below poverty level

^4 ^Percent of persons age 25+ years with less than 12 years of education in census tract

^5 ^Median household income in census tract

^6 ^SES index = summation of quartiles of poverty + high school graduate + median income

^7 ^Colorectal cancer-directed surgery with or without radiation. NC = Non-cancer directed; GT Part/LT Tot = greater than partial/less than total; Surgery, NOS = surgery, not otherwise specified

^8 ^Any radiation = beam, implants, isotopes, or combination; unknown = refused, recommended but don't know if administered, and unknown if administered

^9 ^Chemotherapy assessed using data from the physician supplier files

^10 ^For stage III colon cancer, guideline treatment is receipt of surgery and chemotherapy; for stages II and III rectal cancer, guideline treatment is receipt of surgery, chemotherapy, and radiation

^11 ^Comorbidity measured with Charlson Index using data from physician supplier and inpatient hospitalization files

### Analytical Variables

Race/ethnicity was classified according to the categories shown in the tables. These categories were created to be as specific as possible while preserving adequate numbers for stability of estimates. Information on race/ethnicity, age and year of diagnosis, SEER registry, marital status, tumor characteristics, and radiation were obtained from the SEER portion, or Patient Entitlement and Diagnosis Summary File (PEDSF), of the linked database. SEER registry staff members abstract these data elements from medical records at hospitals, doctor's offices and other facilities. In addition to the data originally obtained from SEER, the PEDSF also included data on area-based socioeconomic measures (poverty, percentage of high school graduates, median income) of the 1990 Census tract of residence as well as the urban/rural status of the county of residence.

Patient information on race and ethnicity were extracted from the SEER database, which are primarily based on patients' hospital medical records. Despite variable hospitals practices and policies in recording these information [31,32], the quality of these data have been shown to be generally good [25,33]. Racial/ethnic categories were defined to be consistent with prior publications using SEER data [6,19,34]. Chinese, Japanese, and Filipinos were classified regardless of Hispanic ethnicity. Racial/ethnic groups with fewer than 200 patients were excluded; these groups include American Indians/Alaskan Natives, Native Hawaiians and other Pacific Islanders, and other smaller Asian subgroups. Patients coded in the SEER data as Hispanic on the basis of surname only were not classified as Hispanic, because of prior research showing misclassification of surname-only classifications.

Census socioeconomic variables corresponding to the tract of patients' addresses at diagnosis are available in the linked SEER-Medicare data; more detailed geographic level measures are not available. Social epidemiologists have noted that while the sizes of the populations covered by census tract units are large and thus limited for conceptualizations of neighborhoods, they have also acknowledged that in the absence of better data, this level of geographical aggregation is acceptable and standard practice for examining neighborhood SES [35,36]. In fact, prior publications of national SEER data have examined contextual SES at an even broader level of geography [37-41]. We examined measures of census tract SES (poverty, education, and income, as defined in Table 1) and also computed a composite measure (SES index) summing the quartiles of each individual measure. Quartiles are based on distributions of the entire study population. This SES index ranged from 1–4, with 1 representing the highest SES quartile and 4 representing the lowest SES. Overall, there were 2937 (7%) patients with missing SES data, ranging from 7% among Whites to 20% among Filipinos; it is likely that these patients could not be reliability geocoded to a census tract. In the absence of individual-level SES data in the cancer registry and Medicare databases, area-based SES measures are useful in this study for examining the impact of neighborhood socioeconomic status on cancer survival. These measures have been shown to be independently predictive of an array of health outcomes [36,42-54]. In this study, the area-based SES measures are not meant to serve as a proxy for individual-level SES.

We used claims information on inpatient hospitalizations and physicians visits occurring 12 months before and 4 months after diagnosis for classification of comorbidities using the Charlson comorbidity index [55,56]. The Charlson index assigns weights from 1 to 6 corresponding to disease severity, for 19 medical conditions. The weights are then summed to provide an overall score. For this analysis we excluded metastatic and *in-situ *colon and rectum cancers from the comorbidity index and adapted standard inclusion criteria, which specifies that physician admissions are valid comorbidities only if they occur on two or more claims more than 30 days apart [30,57,58]. Patients with no admissions records during this period were considered to have had no comorbidities. Medicare only requires claims data for care covered by fee-for-service, or indemnity, insurance; thus claims data for HMO enrollees were often not available in this database. We conducted the analyses that incorporate comorbidity information excluding those aged 65 at diagnosis [59] and did not find that our results changed from those including this group. As the numbers of patients with two or more comorbidities in most of the racial/ethnic groups are small (Table 1), we analyzed comorbidity in subsequent models as none versus any.

SEER-modified AJCC (American Joint Committee on Cancer) staging scheme, 3rd edition (1988+) was used to define tumor stage at diagnosis. AJCC stage is determined based on detailed information in SEER on extent of disease – tumor size, nodal involvement, and extent of metastasis. SEER registries collect information regarding treatment administered during the first four months post-diagnosis. For surgery, patients were classified as having received partial colectomy if they underwent segmental resection (cecectomy, appendectomy, sigmoidectomy, partial resection of transverse colon and flexures, ileocolectomy, enterocolectomy, and partial/subtotal colectomy), laser surgery, polypectomy, cryosurgery, or fulguration; and total/hemicolectomy if they received hemicolectomy or greater, all right/left and portion of transverse colectomy, total colectomy, non-specified colectomy, or colectomy plus partial/total removal of other organs. Information on receipt of radiation was obtained from SEER records; prior research has shown that SEER data alone on radiation is >95% complete [60,61]. Chemotherapy was obtained from physician claims data (for a period of six months after diagnosis [62]) and coded into the categories shown in Table 1. Patients without physician claims data during this time period were coded as not having received chemotherapy. Warren et al previously documented that physician claims alone captures about 70% of chemotherapy administration for colon and rectal cancers [62]. In this cohort, the vast majority of patients (92%) receiving chemotherapy received 5-fluorouracil. We also defined treatment as receiving guideline therapy according to the NIH consensus conference standards, which includes surgery plus adjuvant chemotherapy for stage III colon cancer and surgery plus adjuvant chemotherapy and radiation for stages II and III rectal cancer [63-67]. We have included each of these treatment modalities as separate variables into the models to assess their independent impact on survival; this practice is consistent with prior analyses based on cancer registry data [5,68-70]. In addition, within stage III colon and stages II and III rectal cancer, we have adjusted for one single variable representing receipt of guideline therapy.

### Follow-up

Date and cause of death information were obtained from SEER. Registries conduct active and passive follow-up of cancer patients for vital status using linkages with state and national death indices, Centers for Medicare and Medicaid Services files, driver's license registration files, voter registration files, Social Security Administration files, national credit agency records, and other databases, as well as contact with patients, hospitals and physicians' offices. Through linkage of the SEER-Medicare data to a more recent version of the SEER public use data, patients in this analysis were followed for vital status through December 31, 1999, which is also the date of censoring for patients who were last known to be alive. Underlying cause of death was abstracted from death certificates, and deaths assigned International Classification of Disease – Ninth Revision (ICD-9) codes 153.0–154.1 were identified as due to colon or rectal cancer.

### Statistical Analyses

The Mantel-Haenszel chi-square test was used to compute p-values for differences in distribution of patient and clinical characteristics across racial/ethnic groups. Five-year cause-specific survival probabilities and associated 95% confidence intervals (CI) were computed based on Kaplan-Meier survival curves. This analysis focuses on cause-specific (colorectal) survival as the outcome; however, racial/ethnic patterns were generally similar with the two types of outcomes – all-cause survival and colorectal specific-survival. Cox proportional hazards regression was used to compute relative rates of dying of colorectal cancer specifically and of all causes. Proportionality of hazards was checked graphically and confirmed use of the Cox regression models. Cells with less than five cases are not shown for privacy purposes. Models were computed with parameters representing each non-White racial/ethnic group to assess mortality difference compared to non-Hispanic Whites (Tables 2 (unadjusted survival), 3 (cause-specific mortality), and 4 (all-cause mortality)); independent variables were included in turn to assess the relative impact on the racial/ethnic differences. Models were also computed for each racial/ethnic group (Tables 5 (cause-specific mortality) and 6 (all-cause mortality)) to assess the relative effects of the sociodemographic and clinical characteristics on mortality within groups. To assess whether differences in the parameter estimates were significant across racial/ethnic groups, we compared the log likelihood ratios for a main effects model with race/ethnicity and the independent prognostic factors with a model containing an interaction term of race/ethnicity and the prognostic factor of interest.

**Table 2:** Unadjusted survival estimates, by sex and race/ethnicity (N = 41,901), SEER, 1992–1996

**Survival**	**Non-Hispanic White**	**Non-Hispanic Black**	**Hispanic**	**Chinese**	**Japanese**	**Filipino**
**Males (N = 19,979)**	**16,213**	**1,356**	**1,032**	**498**	**601**	**279**
Colorectal cancer deaths, N (%)	5119 (32)	531 (39)	375 (36)	154 (31)	157 (26)	88 (32)
5-Year colorectal survival (95% CI)	0.65 (0.64–0.65)	0.55 (0.52–0.58)	0.60 (0.56–0.63)	0.67 (0.62–0.71)	0.71 (0.67–0.75)	0.64 (0.58–0.70)
All cause deaths, N (%)	9116 (56)	854 (63)	569 (55)	252 (51)	293 (49)	153 (55)
5-Year all cause survival (95% CI)	0.45 (0.44–0.46)	0.37 (0.35–0.40)	0.46 (0.41–0.49)	0.51 (0.46–0.56)	0.53 (0.49–0.57)	0.47 (0.41–0.53)
**Females (N = 21,922)**	**17,951**	**1,828**	**1,029**	**383**	**515**	**216**
Colorectal cancer deaths, N (%)	5865 (33)	723 (40)	33 (32)	132 (34)	146 (28)	66 (31)
5-Year colorectal survival (95% CI)	0.64 (0.63–0.65)	0.55 (0.53–0.58)	0.64 (0.60–0.67)	0.62 (0.57–0.67)	0.69 (0.64–0.73)	0.68 (0.60–0.74)
All cause deaths, N (%)	9722 (54)	1125 (62)	538 (52)	185 (48)	226 (44)	99 (46)
5-Year all cause survival (95% CI)	0.47 (0.47–0.48)	0.39 (0.37–0.41)	0.48 (0.45–0.51)	0.52 (0.47–0.57)	0.57 (0.52–0.61)	0.54 (0.46–0.60)

**Table 3:** Relative rates (and 95% confidence intervals) of dying of colorectal cancer for each racial/ethnic group (relative to non-Hispanic Whites), SEER, 1992–1996

**Variables in model**	**Non-Hispanic Black ** **N = 3,009**	**Hispanic ** **N = 1,951**		**Chinese ** **N = 851**	**Japanese ** **N = 1,094**	**Filipino ** **N = 479**
		**Male ** **N = 1,032**	**Female ** **N = 1,029**			
					
1. Race/ethnicity	1.33 (1.26–1.42)	1.16 (1.04–1.29)	0.98 (0.88–1.10)	0.98 (0.87–1.11)	0.79 (0.70–0.88)	0.99 (0.84–1.16)
2. Race/ethnicity + sociodemographics^1^	1.32 (1.24–1.41)	1.12 (1.00–1.26)	1.02 (0.91–1.15)	1.06 (0.93–1.19)	0.94 (0.82–1.09)	1.09 (0.92–1.30)
3. Race/ethnicity + sociodemographics & disease characteristics^2^	1.26 (1.18–1.34)	1.01 (0.90–1.13)	0.93 (0.82–1.04)	1.02 (0.90–1.16)	0.97 (0.84–1.12)	0.96 (0.81–1.13)
4. Race/ethnicity + sociodemographics, disease characteristics, & treatment^3^	1.21 (1.14–1.29)	1.01 (0.90–1.14)	0.94 (0.83–1.06)	0.97 (0.86–1.10)	0.98 (0.85–1.13)	0.91 (0.76–1.07)
5. Race/ethnicity + sociodemographics, disease characteristics, treatment, & education	1.16 (1.09–1.24)	0.98 (0.87–1.10)	0.92 (0.81–1.04)	0.95 (0.84–1.08)	0.96 (0.83–1.11)	0.88 (0.74–1.05)
6. Race/ethnicity + sociodemographics, disease characteristics, treatment, & income	1.17 (1.09–1.25)	0.99 (0.89–1.12)	0.93 (0.83–1.05)	0.96 (0.85–1.09)	0.98 (0.85–1.12)	0.90 (0.76–1.07)
7. Race/ethnicity + sociodemographics, disease characteristics, treatment, & poverty	1.16 (1.09–1.25)	0.99 (0.88–1.11)	0.93 (0.83–1.05)	0.96 (0.84–1.08)	0.98 (0.85–1.12)	0.90 (0.76–1.06)
8. Race/ethnicity + sociodemographics, disease characteristics, treatment, & comorbidities	1.21 (1.14–1.29)	1.01 (0.90–1.13)	0.94 (0.83–1.05)	0.97 (0.86–1.10)	0.98 (0.85–1.13)	0.91 (0.77–1.08)

^1 ^sociodemographic factors = age and marital status at diagnosis, sex, year of diagnosis, and SEER region

^2 ^disease characteristics = stage, grade, site

^3 ^treatment = surgery, radiation, chemotherapy

**Table 4:** Relative rates (and 95% confidence intervals) of dying of any cause for each racial/ethnic group (relative to non-Hispanic Whites), SEER, 1992–1996

**Variables in model**	**Non-Hispanic Black **	**Hispanic **		**Chinese**	**Japanese**	**Filipino**
	**N = 3,009**	**N = 1,951**	**N=851**	**N=1,094**	**N=479**
		**Male N = 1,032**	**Female N = 1,029**			
					
1. Race/ethnicity	1.23 (1.18–1.30)	1.01 (0.92–1.10)	0.97 (0.89–1.07)	0.88 (0.80–0.97)	0.78 (0.72–0.85)	0.95 (0.84–1.08)
2. Race/ethnicity + sociodemographics^1^	1.23 (1.17–1.29)	1.03 (0.94–1.13)	1.03 (0.94–1.13)	0.95 (0.86–1.05)	0.95 (0.85–1.06)	1.05 (0.92–1.20)
3. Race/ethnicity + sociodemographics & disease characteristics^2^	1.18 (1.13–1.24)	0.96 (0.88–1.06)	0.95 (0.87–1.04)	0.92 (0.83–1.02)	0.97 (0.87–1.09)	0.97 (0.85–1.11)
4. Race/ethnicity + sociodemographics, disease characteristics, & treatment^3^	1.14 (1.08–1.20)	0.97 (0.89–1.06)	0.96 (0.87–1.05)	0.88 (0.80–0.97)	0.98 (0.88–1.09)	0.93 (0.81–1.06)
5. Race/ethnicity + sociodemographics, disease characteristics, treatment, & education	1.09 (1.04–1.15)	0.93 (0.84–1.02)	0.93 (0.85–1.02)	0.86 (0.78–0.95)	0.96 (0.86–1.07)	0.90 (0.78–1.03)
6. Race/ethnicity + sociodemographics, disease characteristics, treatment, & income	1.09 (1.04–1.16)	0.94 (0.86–1.04)	0.95 (0.86–1.04)	0.87 (0.79–0.96)	0.98 (0.88–1.09)	0.93 (0.81–1.06)
7. Race/ethnicity + sociodemographics, disease characteristics, treatment, & poverty	1.08 (1.03–1.14)	0.94 (0.86–1.03)	0.95 (0.86–1.04)	0.87 (0.78–0.96)	0.97 (0.87–1.09)	0.92 (0.80–1.05)
8. Race/ethnicity + sociodemographics, disease characteristics, treatment, & comorbidities	1.14 (1.09–1.20)	0.96 (0.88–1.05)	0.95 (0.87–1.05)	0.91 (0.82–1.00)	0.99 (0.89–1.11)	0.95 (0.83–1.08)

^1 ^sociodemographic factors = age and marital status at diagnosis, sex, year of diagnosis, and SEER region

^2 ^disease characteristics = stage, grade, site

^3 ^treatment = surgery, radiation, chemotherapy

**Table 5:** Adjusted^1 ^relative rates (and 95% confidence intervals) of dying of colorectal cancer associated with selected demographic and clinical characteristics, within race/ethnic group, SEER, 1992–1996, N = 40,209^2^

**Characteristic (referent group)**	**Non-Hispanic White **			**Non-Hispanic Black **			**Hispanic **			**Chinese**			**Japanese**			**Filipino N = 479**		
	**N = 32,825**	**N = 3,009**	**N = 851**	**N = 32,825**	**N = 1,094**	**N = 479**
	
	Adjusted RR	95% CI		Adjusted RR	95% CI		Adjusted RR	95% CI		Adjusted RR	95% CI		Adjusted RR	95% CI		Adjusted RR	95% CI	
Age (65–69)																		
70–74	1.08	1.02	-1.15	1.28	1.09	-1.51	1.21	0.97	-1.51	1.14	0.76	-1.70	1.25	0.89	-1.75	0.76	0.43	-1.32
75–79	1.18	1.11	-1.25	1.28	1.08	-1.52	1.35	1.07	-1.70	1.11	0.74	-1.67	1.62	1.12	-2.33	1.60	0.99	-2.59
80+	1.53	1.44	-1.62	1.35	1.14	-1.59	1.79	1.43	-2.23	1.99	1.34	-2.95	1.77	1.24	-2.53	1.60	1.00	-2.57
Gender (female)																		
Male	1.06	1.02	-1.11	1.02	0.90	-1.15	1.25	1.05	-1.48	1.17	0.90	-1.54	0.94	0.73	-1.21	1.26	0.86	-1.85
Marital status (ever married)																		
Single	1.14	1.09	-1.19	0.98	0.86	-1.12	1.24	1.04	-1.46	1.33	1.00	-1.76	1.21	0.92	-1.60	0.91	0.62	-1.34
Diagnosis year (1992–1993)																		
1994–1996	0.99	0.95	-1.03	0.94	0.84	-1.06	1.03	0.88	-1.21	1.11	0.85	-1.45	0.86	0.67	-1.10	0.94	0.66	-1.35
AJCC stage (I)																		
II	2.33	2.12	-2.55	2.65	2.02	-3.47	1.93	1.37	-2.72	3.74	1.94	-7.23	2.37	1.32	-4.24	2.60	1.14	-5.90
III	5.62	5.13	-6.14	4.63	3.54	-6.04	3.89	2.77	-5.47	8.15	4.25	-15.62	7.34	4.27	-12.61	4.52	2.07	-9.85
IV	23.69	21.67	-25.90	20.36	15.70	-26.40	17.04	12.23	-23.74	35.12	18.14	-68.02	41.39	23.68	-72.36	18.22	8.36	-39.70
Unknown	3.63	3.25	-4.06	3.25	2.40	-4.42	2.76	1.76	-4.34	3.77	1.81	-7.85	2.45	1.15	-5.23	3.49	1.39	-8.73
Grade (well)																		
Moderate	1.22	1.12	-1.32	1.21	0.95	-1.54	1.28	0.91	-1.80	2.46	1.24	-4.91	0.90	0.43	-1.88	0.93	0.43	-2.01
Poor/None	1.86	1.70	-2.03	1.90	1.45	-2.49	1.98	1.38	-2.85	3.65	1.79	-7.45	1.25	0.58	-2.72	1.23	0.53	-2.84
Unknown	1.29	1.17	-1.43	1.26	0.95	-1.68	1.20	0.80	-1.79	1.92	0.88	-4.23	0.69	0.29	-1.62	0.28	0.10	-0.80
Site (rectal)																		
Distal	0.90	0.84	-0.95	0.86	0.72	-1.03	0.91	0.72	-1.16	0.98	0.69	-1.37	0.99	0.70	-1.39	1.11	0.70	-1.77
Proximal	1.01	0.96	-1.06	0.91	0.77	-1.07	0.89	0.72	-1.11	1.04	0.74	-1.46	0.89	0.63	-1.26	1.35	0.83	-2.19
Surgery (none/NC direct)																		
GT Partial LT Total	0.30	0.28	-0.32	0.28	0.23	-0.33	0.30	0.23	-0.39	0.25	0.16	-0.38	0.18	0.11	-0.29	0.19	0.11	-0.33
Partial	0.27	0.24	-0.32	0.30	0.20	-0.44	0.17	0.09	-0.33	0.66	0.29	-1.49	0.13	0.05	-0.32	0.66	0.22	-1.96
Total	0.37	0.34	-0.40	0.41	0.33	-0.51	0.38	0.28	-0.52	0.41	0.24	-0.70	0.21	0.12	-0.37	0.16	0.08	-0.33
Radiation (none)																		
Any	0.98	0.92	-1.05	0.78	0.63	-0.97	1.00	0.77	-1.29	0.99	0.62	-1.59	1.21	0.78	-1.88	0.79	0.41	-1.51
Chemotherapy (none)																		
Any	0.89	0.83	-0.95	0.64	0.49	-0.85	0.75	0.60	-0.94	0.76	0.52	-1.10	0.91	0.57	-1.46	1.14	0.67	-1.94
SEER region(SFBA)																		
Atlanta	0.97	0.88	-1.08	0.75	0.60	-0.94	0.25	0.06	-1.02	1.20	0.28	-5.13	-		-			-
Connecticut	0.97	0.90	-1.04	0.81	0.60	-1.09	0.51	0.32	-0.82	1.58	0.21	-12.07	0.00	0.00	-	1.64	0.34	-7.94
Detroit	0.98	0.91	-1.06	0.91	0.76	-1.09	1.05	0.48	-2.30	0.31	0.04	-2.33	4.24	0.53	-33.64	9.86	1.09	-89.23
Hawaii	0.84	0.68	-1.03	1.43	0.35	-5.89	1.12	0.16	-8.15	0.66	0.41	-1.04	1.21	0.80	-1.83	0.75	0.48	-1.17
Iowa	0.94	0.87	-1.03	0.92	0.54	-1.58	1.32	0.58	-3.04	-		-	1.54	0.20	-11.74	-		-
LA	0.98	0.91	-1.05	0.84	0.70	-1.01	0.74	0.60	-0.91	1.24	0.92	-1.66	1.32	0.87	-2.02	1.26	0.81	-1.98
New Mexico	1.13	1.00	-1.28	0.93	0.43	-2.02	0.88	0.67	-1.17			-			-	-		-
Seattle	1.02	0.94	-1.11	0.95	0.67	-1.35	0.74	0.36	-1.55	0.93	0.48	-1.82	0.99	0.53	-1.85	0.67	0.25	-1.81
Utah	1.12	1.00	-1.26	1.76	0.56	-5.54	0.86	0.47	-1.61			-	0.96	0.23	-4.10	-	-	
Median income quartiles (1^st ^highest SES)																		
2^nd^	0.91	0.86	-0.98	0.88	0.57	-1.34	0.76	0.53	-1.09	0.87	0.51	-1.49	1.02	0.68	-1.53	1.62	0.94	-2.79
3^rd^	0.95	0.87	-1.04	0.88	0.55	-1.41	0.77	0.50	-1.18	1.18	0.64	-2.19	1.05	0.63	-1.75	1.89	0.88	-4.09
4^th^	0.92	0.82	-1.03	0.96	0.59	-1.58	0.87	0.53	-1.42	1.07	0.50	-2.29	1.59	0.82	-3.08	2.54	1.02	-6.33
Unknown	6.31	0.89	-44.94	0.96	0.64	-1.42	0.70	0.49	-1.02	1.46	0.86	-2.47	0.91	0.57	-1.45	2.18	0.87	-5.45
Below poverty quartiles (1^st ^highest SES)																		
2^nd^	1.05	0.99	-1.12	0.77	0.51	-1.17	0.94	0.66	-1.35	1.50	0.89	-2.51	1.03	0.68	-1.56	1.39	0.67	-2.86
3^rd^	1.08	1.00	-1.16	0.77	0.49	-1.21	1.06	0.70	-1.61	1.38	0.70	-2.70	1.18	0.71	-1.96	1.27	0.59	-2.70
4^th^	1.06	0.96	-1.17	0.85	0.52	-1.37	1.18	0.73	-1.90	1.59	0.72	-3.50	0.95	0.51	-1.76	0.91	0.34	-2.38
Unknown	-		-	-		-	-		-	-		-	-		-	-		-
Non-HS quartiles (1^st ^highest SES)																		
2^nd^	1.09	1.02	-1.15	1.28	0.89	-1.83	0.79	0.56	-1.11	0.63	0.39	-1.01	0.82	0.54	-1.25	1.65	0.72	-3.77
3^rd^	1.08	1.01	-1.16	1.32	0.90	-1.93	0.74	0.51	-1.06	0.73	0.42	-1.28	0.85	0.54	-1.33	0.93	0.38	-2.25
4^th^	1.14	1.04	-1.24	1.32	0.90	-1.95	0.79	0.53	-1.17	0.84	0.46	-1.53	0.98	0.63	-1.54	1.14	0.49	-2.63
Unknown	0.17	0.02	-1.21	-		-	-		-	-		-	-		-	-		-
Comorbidity (none)																		
One or more	1.08	1.03	-1.12	1.09	0.96	-1.23	1.07	0.90	-1.28	1.05	0.75	-1.48	0.98	0.73	-1.33	1.28	0.82	-2.00

^1 ^Adjusted for the other factors in the table.

^2^Excludes patients with unknown marital status and/or unknown radiation

**Table 6:** Adjusted^1 ^relative rates (and 95% confidence intervals) of dying of any cause associated with selected demographic and clinical characteristics, within race/ethnic group, SEER, 1992–1996, N = 40,209^2^

**Characteristic (referent group)**	**Non-Hispanic White**			**Non-Hispanic Black **			**Hispanic**			**Chinese **			**Japanese **			**Filipino **		
	**N = 32,825**	**N = 3,009**	**N = 1,951**	**N = 851**	**N = 1,094**	**N = 479**
	
	Adjusted RR	95% CI		Adjusted RR	95% CI		Adjusted RR	95% CI		Adjusted RR	95% CI		Adjusted RR	95% CI		Adjusted RR	95% CI	
Age (65–69)																		
70–74	1.17	1.12	-1.23	1.28	1.12	-1.47	1.27	1.06	-1.53	1.32	0.94	-1.87	1.25	0.96	-1.63	0.84	0.53	-1.33
75–79	1.43	1.36	-1.50	1.39	1.21	-1.60	1.50	1.25	-1.81	1.42	1.01	-1.99	1.68	1.28	-2.22	2.01	1.35	-2.99
80+	2.04	1.95	-2.13	1.76	1.54	-2.01	2.07	1.74	-2.47	2.69	1.94	-3.74	2.32	1.77	-3.03	2.20	1.51	-3.21
Gender (female)																		
Male	1.22	1.18	-1.26	1.12	1.01	-1.24	1.24	1.09	-1.42	1.38	1.11	-1.72	1.26	1.04	-1.53	1.28	0.95	-1.73
Marital status (ever married)																		
Single	1.22	1.18	-1.26	1.10	0.99	-1.22	1.24	1.08	-1.41	1.27	1.02	-1.59	1.23	1.00	-1.52	0.93	0.69	-1.25
Diagnosis year (1992–1993)																		
1994–1996	0.98	0.95	-1.01	0.94	0.86	-1.04	1.00	0.88	-1.14	1.01	0.82	-1.25	0.96	0.79	-1.16	1.02	0.77	-1.36
AJCC stage (I)																		
II	1.34	1.27	-1.41	1.67	1.41	-1.97	1.32	1.06	-1.65	1.74	1.19	-2.55	1.23	0.90	-1.66	1.80	1.09	-2.96
III	2.26	2.15	-2.38	2.25	1.90	-2.67	2.14	1.71	-2.69	2.94	2.00	-4.31	2.41	1.81	-3.22	2.54	1.56	-4.13
IV	7.69	7.29	-8.11	7.81	6.60	-9.23	7.06	5.65	-8.82	10.10	6.76	-15.08	11.22	8.12	-15.51	7.65	4.65	-12.59
Unknown	1.73	1.61	-1.85	1.81	1.48	-2.22	1.88	1.39	-2.55	2.42	1.53	-3.84	1.27	0.81	-1.98	2.15	1.17	-3.94
Grade (well)																		
Moderate	1.11	1.05	-1.18	0.98	0.82	-1.16	1.47	1.13	-1.91	1.27	0.81	-1.97	0.91	0.58	-1.42	1.23	0.67	-2.26
Poor/None	1.52	1.43	-1.62	1.54	1.27	-1.88	2.13	1.60	-2.84	1.87	1.17	-3.01	1.23	0.75	-2.03	1.46	0.75	-2.85
Unknown	1.16	1.08	-1.24	1.12	0.92	-1.38	1.32	0.96	-1.80	1.10	0.65	-1.88	0.81	0.47	-1.38	0.70	0.34	-1.45
Site (rectal)																		
Distal	0.94	0.90	-0.98	0.96	0.83	-1.11	1.00	0.83	-1.20	1.03	0.79	-1.35	1.01	0.79	-1.29	1.02	0.71	-1.48
Proximal	0.99	0.95	-1.03	0.98	0.86	-1.12	0.98	0.83	-1.17	1.02	0.77	-1.34	0.91	0.70	-1.17	1.53	1.06	-2.21
Surgery (none/NC direct)																		
GT Partial LT Total	0.33	0.31	-0.35	0.30	0.25	-0.35	0.30	0.24	-0.38	0.34	0.24	-0.48	0.21	0.15	-0.31	0.22	0.14	-0.34
Partial	0.39	0.36	-0.42	0.36	0.28	-0.46	0.31	0.21	-0.44	0.67	0.39	-1.16	0.25	0.16	-0.42	0.82	0.41	-1.66
Total	0.38	0.36	-0.41	0.44	0.36	-0.53	0.39	0.30	-0.51	0.46	0.29	-0.72	0.26	0.17	-0.40	0.19	0.10	-0.34
Radiation (none)																		
Any	0.93	0.88	-0.99	0.78	0.65	-0.95	0.99	0.80	-1.22	1.06	0.72	-1.55	0.99	0.69	-1.40	0.76	0.45	-1.30
Chemotherapy (none)																		
Any	0.81	0.77	-0.86	0.61	0.48	-0.78	0.69	0.57	-0.84	0.78	0.57	-1.08	0.86	0.57	-1.30	0.94	0.59	-1.51
SEER region (SFBA)																		
Atlanta	0.99	0.91	-1.07	0.85	0.71	-1.02	0.53	0.23	-1.21	1.97	0.69	-5.67	-		-	0.00	0.00	-
Connecticut	1.00	0.95	-1.06	1.07	0.85	-1.34	0.69	0.49	-0.98	2.65	0.80	-8.83	0.00	0.00	-	1.15	0.26	-5.11
Detroit	0.97	0.92	-1.03	1.10	0.95	-1.28	1.04	0.53	-2.07	0.36	0.09	-1.51	6.60	1.51	-28.80	2.35	0.28	-19.78
Hawaii	0.80	0.68	-0.94	0.74	0.18	-3.01	1.60	0.51	-5.08	0.85	0.60	-1.20	1.20	0.86	-1.67	0.94	0.67	-1.32
Iowa	0.92	0.86	-0.98	0.93	0.60	-1.42	1.35	0.71	-2.58	-		-	0.91	0.12	-6.73	-		-
LA	1.04	0.99	-1.10	1.00	0.86	-1.16	0.88	0.74	-1.04	1.23	0.97	-1.56	1.34	0.96	-1.89	1.58	1.11	-2.26
New Mexico	1.01	0.92	-1.11	1.05	0.58	-1.89	0.84	0.67	-1.05	1.04	0.13	-8.20			-	-		-
Seattle	0.99	0.93	-1.05	1.05	0.78	-1.40	0.65	0.36	-1.18	0.98	0.58	-1.66	1.11	0.67	-1.85	0.82	0.41	-1.64
Utah	1.02	0.93	-1.11	1.13	0.36	-3.54	0.85	0.52	-1.38			-	1.00	0.36	-2.84	-	-	
Median income quartiles (1^st ^highest SES)																		
2^nd^	0.94	0.90	-0.99	0.90	0.65	-1.25	0.86	0.65	-1.15	0.95	0.61	-1.48	0.91	0.68	-1.21	1.50	0.99	-2.27
3^rd^	0.96	0.90	-1.02	1.01	0.71	-1.46	0.98	0.70	-1.37	1.17	0.70	-1.97	0.87	0.60	-1.25	1.79	1.02	-3.15
4^th^	0.92	0.85	-1.01	1.05	0.71	-1.54	1.14	0.77	-1.67	1.28	0.69	-2.39	1.02	0.63	-1.65	1.82	0.94	-3.54
Unknown	7.10	1.00	-50.37	1.11	0.81	-1.51	0.86	0.64	-1.17	1.62	1.06	-2.47	1.00	0.70	-1.44	1.97	1.03	-3.76
Below poverty quartiles (1^st ^highest SES)																		
2^nd^	1.04	1.00	-1.09	0.80	0.57	-1.10	0.94	0.70	-1.27	1.34	0.88	-2.03	1.27	0.94	-1.71	1.83	1.07	-3.14
3^rd^	1.06	1.00	-1.13	0.76	0.54	-1.08	1.09	0.78	-1.51	1.15	0.67	-1.96	1.21	0.84	-1.76	1.46	0.81	-2.61
4^th^	1.09	1.02	-1.18	0.82	0.57	-1.19	1.05	0.71	-1.55	1.25	0.66	-2.35	1.23	0.77	-1.95	1.33	0.66	-2.70
Unknown	-		-	-		-	-		-	-		-	-		-	-		-
Non-HS quartiles (1^st ^highest SES)																		
2^nd^	1.06	1.02	-1.11	1.17	0.89	-1.55	0.80	0.61	-1.04	0.91	0.62	-1.35	0.95	0.69	-1.31	1.05	0.58	-1.89
3^rd^	1.09	1.03	-1.15	1.28	0.96	-1.72	0.70	0.52	-0.94	1.00	0.63	-1.58	0.95	0.68	-1.34	0.69	0.37	-1.28
4^th^	1.13	1.06	-1.20	1.18	0.88	-1.59	0.79	0.58	-1.08	1.09	0.66	-1.79	1.20	0.85	-1.68	0.89	0.50	-1.58
Unknown	0.16	0.02	-1.13	-		-	-		-	-		-	-		-	-		-
Comorbidity (none)																		
One or more	1.50	1.46	-1.55	1.41	1.28	-1.55	1.34	1.17	-1.53	1.35	1.05	-1.74	1.53	1.25	-1.87	1.73	1.26	-2.37

^1 ^Adjusted for the other factors in the table.

^2^Excludes patients with unknown marital status and/or unknown radiation

Human subjects issues were reviewed and approved by the Northern California Cancer Center Institutional Review Board.

## Results

### Characteristics of study population

Table 1 shows the distribution of patient and clinical characteristics, by race/ethnicity. There were proportionally fewer males than females among Blacks and more males than females among Asians. Two-thirds of Blacks, about half of Hispanics, and one-third of Chinese lived in lower SES Census tracts. Whites and Japanese were least likely to be living in the lowest SES tracts. Chinese, Japanese, and Filipinos were most likely to be married, while Blacks were the least likely.

Whites and Blacks had higher proportions of proximal colon tumors than rectal cancers. In contrast, Filipinos had more distal and rectal tumors. Blacks, Hispanics, and Filipinos were more likely than other racial/ethnic groups to be diagnosed with stage III and IV disease, and Whites, Hispanics, and Chinese were more likely to be diagnosed with poorly or undifferentiated tumors. Proportionally more Blacks received no surgery or no cancer directed surgery (14%). Only about 10% overall received radiation treatment, varying from 7% among Blacks to 11% among Hispanics. From 4–5% of Blacks and Japanese to 13% of Hispanics had chemotherapy. Relatively fewer Blacks and Japanese received guideline treatment for stage III colon cancer compared to Hispanics and Chinese, and relatively fewer Blacks, Japanese, and Filipinos received guideline treatment for stages II and III rectal cancer. Approximately one-third of Whites, Blacks, and Hispanics had one or more comorbid conditions, compared to smaller percentages in the other groups. Proportionally more Blacks had two or more comorbid conditions than the other groups, particularly the three Asian subgroups.

Table 2 shows unadjusted survival estimates by sex and race/ethnicity. Both Black males and females experienced decreased 5-year survival compared to other groups. Other notable racial/ethnic variations in survival include decreased disease-specific survival among Hispanic males. Some heterogeneity in survival among Asian subgroups was evident: Filipino males and Chinese females experienced slightly decreased disease-specific and overall survival.

### Factors associated with survival/mortality

#### Race/ethnic mortality relative to Whites

Table 3 shows the relative rates of dying of colorectal cancer for each racial/ethnic group compared to Whites. Racial/ethnic patterns, relative to Whites, were similar between males and females and are thus combined, with the exception of Hispanics, for whom gender differences, in relation to Whites, were evident and thus presented separately. Compared to Whites, colorectal cancer-specific mortality rates were 33% higher among Blacks and 16% higher among Hispanic males. Mortality rates among Japanese were lower than Whites, while mortality among Hispanic females, Chinese and Filipinos was comparable to Whites. Among Blacks, adjusting for disease characteristics (primarily stage) reduced the hazard ratio from 1.32 to 1.26, and while adjusting independently for education level, poverty, and income further reduced the hazard ratio, mortality among Blacks was still 16% significantly higher than Whites. Among Hispanic males, adjustment for disease characteristics (primarily stage) appeared to account for their higher mortality compared to Whites. Among Japanese, the significantly lower mortality rate compared to Whites was partially attributable to disease characteristics but mostly to sociodemographic factors (primarily SEER region). Comorbidity did not affect racial/ethnic differences in colorectal cause-specific mortality rates.

Table 4 shows the relative rates of dying of any cause for each racial/ethnic group compared to Whites. The 23% higher rate of death among Blacks compared to Whites was partially attributable to disease characteristics, socioeconomic status, and treatment (to a lesser extent), although the all-cause mortality rate among Blacks remained significantly higher than the rate among Whites even after adjusting for all of these factors simultaneously. Comorbidity did not affect the all-cause mortality difference between Black and White colorectal cancer patients. Chinese and Japanese experienced significantly lower all-cause mortality compared to Whites; for both groups, their more favorable mortality rates were attributable to sociodemographic factors. There were no differences in all-cause mortality between Hispanics, Filipinos, and Whites.

Focusing on those stages of cancer for which guidelines exist for standard of care (i.e., stage III colon and stages II/III rectal cancer, data not shown), Blacks had 10% higher rate of death due to all causes compared to Whites (hazard ratio = 1.10, 95% CI 1.00–1.21). This hazard rate ratio ranged from 1.05 to 1.07 and was statistically non-significant after including each of the SES variables (education, income, or poverty) into the model. Similarly, for colorectal cancer specific survival, Blacks had 15% higher rate of death compared to Whites (hazard ratio = 1.15, 95% CI 1.03–1.28), which was completely accounted for by adjusting for each SES variable. Adjusting for guideline treatment did not alter the small difference in all-cause or colorectal specific survival between Blacks and Whites.

#### Race/ethnic-specific mortality

Proportional hazards modeling was conducted separately for each racial/ethnic group and the adjusted relative rates are shown in Tables 5 (disease-specific mortality) and 6 (all-cause mortality). For disease-specific mortality, among Whites, older age, male gender, single marital status, advanced stage, advanced grade, rectal site (compared to distal), having no surgery, having no chemotherapy, residence in New Mexico or Utah (relative to San Francisco Bay Area (SFBA)), and having one or more comorbidities were all independently associated with higher mortality following colorectal cancer diagnosis. Only the SES variable education showed a statistically significant gradient of association with mortality among Whites. Among Blacks, higher mortality was seen among patients of older age, advanced stage and grade, who had no surgery, no radiation, or no chemotherapy. None of the SES variables were significantly associated with mortality among Blacks, although poverty showed non-significantly protective effects, and higher education showed non-significantly adverse effects. Among Hispanics, increased mortality was associated with advanced age, male gender, single marital status, advanced stage and grade, no surgery, and no chemotherapy. Compared to residents in the SFBA, Hispanics in Atlanta, Connecticut, and LA experienced decreased mortality (statistically significant for Connecticut and LA). Among Chinese, statistically significant factors associated with increased mortality were advanced age, single marital status, advanced stage and grade, and no surgery. Among Japanese, the only factors significantly associated with increased mortality were advanced age, advanced stage, and no surgery. Among Filipinos, increased mortality was significantly associated with advanced stage, no surgery, and living in the lowest income neighborhood. None of the SES variables were associated with mortality among Hispanics, Chinese, and Japanese.

Stage had the biggest impact on mortality among all racial/ethnic groups, followed by surgery. However, variations in the importance of the other factors were evident across groups. For example, male gender was associated with increased mortality in Whites and Hispanics, but not among Blacks, Chinese, Japanese, and Filipinos. Single marital status was associated with increased mortality among Whites and Hispanics, and suggested for Chinese and Japanese. Histologic grade was a significant predictive factor of mortality in all groups except for Japanese and Filipinos. Radiation was associated with mortality only among Blacks. Chemotherapy decreased mortality in most groups except Filipinos. Geographic differences in mortality were also apparent, with relatively higher mortality among Whites in New Mexico and Utah; and lower mortality among Hispanics in Atlanta, Connecticut, and LA. Significantly increased mortality was seen with lower income among Filipinos. Little variation in effect was seen among the racial/ethnic groups for poverty, except for slightly increased mortality among Whites and non-significantly protective effect among Blacks. Significant associations with education were seen only among Whites. The tests for significant parameter effects across racial/ethnic-specific colorectal mortality models (ie, interaction of parameter with race/ethnicity) showed that the effects of marital status, stage, and chemotherapy were statistically significant (p < .025, p < .01, p < .01, respectively) across the racial/ethnic groups.

Some differences in factors associated with all-cause mortality (Table 6) compared to cause-specific mortality were evident for each racial/ethnic group, but the most notable difference was the considerably higher impact of comorbidity on mortality, with patients with one or more comorbid conditions having 34% (Hispanics) to 73% (Filipinos) higher mortality than patients without any comorbidities. Another notable difference in all-cause mortality compared to colorectal-specific mortality is the higher mortality among males compared to females across nearly all racial/ethnic groups for all-cause mortality, whereas higher mortality among males for colorectal-cancer specific mortality was observed only among Whites and Hispanics.

## Discussion

Our results, based on a large and representative cohort, show consistently increased mortality (i.e., decreased survival) among Blacks, compared to Whites, that is partially but not completely explained by demographic, disease, treatment, and SES characteristics. This racial/ethnic disparity was evident for mortality due to colorectal cancer as an underlying cause of death, as well as all-cause mortality, although the magnitude of the disparity was larger for colorectal cancer mortality. However, within stage III colon and stages II/III rectal cancer, for which guideline treatments are established, survival differences between Blacks and Whites were considerably smaller and completely explained by SES. We also found decreased survival among Hispanic males that was completely attributable to their being more likely to be diagnosed with advanced stage cancer, and increased survival among Japanese that was due in part to their being more likely to be diagnosed with early stage disease and their place of residence. Our study extends prior population-based racial/ethnic studies of colorectal cancer survival by including additional explanatory factors, including information on comorbidities, radiation, chemotherapy, and area-based SES. In an analysis based on national SEER data, Chien et al. found persistently decreased stage-adjusted survival among certain racial/ethnic groups and postulated that the residual differences were attributable to SES and/or comorbidities [5]. Using SEER data linked to Medicare claims, we were able to evaluate the impact of these factors, plus additional treatment information, on racial/ethnic differences in survival, and found that although SES had some impact on Black-White differences in survival, comorbidities and treatment did not affect the racial/ethnic differences in survival, at least among the Medicare-eligible population. In fact, the presence of comorbidities had minimal impact on colorectal cancer-specific survival in general, and only among non-Hispanic Whites. As expected, comorbidities were more strongly associated with all-cause survival across the racial/ethnic groups. However, we cannot discount the possibility that more detailed measures of comorbidity (e.g., specific conditions or specific combinations of conditions) or a more comprehensive data collection method (e.g., through medical record review [71]) may have more significant impacts on survival. Our results confirm, in a national Medicare population, those from the NCI Black/White Cancer Survival Study conducted in the mid-1980's [11], that the Black-White differential in colorectal survival is partly attributable to stage and SES, but some proportion of the difference remains unexplained. We further found that this pattern was true for all-cause survival as well. A recent review and meta-analysis of 10 studies on colon cancer survival by Du et al. showed that the adjusted hazard ratio for Blacks compared to Whites was 1.14 (1.00–1.29) for all-cause mortality and 1.13 (1.01–1.28) for colon cancer-specific mortality [72]. Among those studies that adjusted simultaneously for age, comorbidity, SES, and treatment, the estimates were 1.16 (1.03–1.32) and 1.30 (1.01–1.67), respectively [72]. These estimates are similar in magnitude to those found in our study, 1.14 (1.09–1.20) and 1.21 (1.14–1.29), respectively, with our results showing slightly tighter confidence intervals.

Thus far, it appears that the only studies showing statistically equal stage-adjusted survival between Blacks and Whites are those done in a VA setting [4,73], suggesting that there may be attributes of the VA system (equal access to screening and treatment, uniform treatment) or its population (similar SES, equal utilization) that are unique from the general population. An analysis comparing lung and colon cancer outcomes between Black and White VA patients who received uniform evaluation and treatment showed that cancer outcomes were similar despite lower SES (individual-level) among Blacks [74]. Shavers and Brown synthesized several studies examining racial/ethnic disparities in colorectal cancer treatment and found that population-based studies tended to find significant racial/ethnic differences in treatment, whereas studies showing no racial/ethnic differences tended to be non-population-based, from a single clinic or locality, or of VA populations [75]. A recent study of a cohort of insured patients found that Black-White differences in survival were explained primarily by stage and receipt of surgery [76]. In a conceptual framework illustrating potential barriers to the receipt of optimal cancer treatment, Shavers and Brown advocated the importance of "structural barriers", which include presence and type of insurance coverage, institutional and geographic factors [75]. In fact, a number of recent, population-based studies have demonstrated a modest but independent effect of surgeon and hospital characteristics [8,77] and of specific types of health care coverage [10], but, to our knowledge, these factors have not been evaluated for their impact on racial/ethnic differentials in survival following colorectal cancer.

The increased survival of Japanese compared to other race/ethnic groups and other Asian subgroups has been noted in other studies [5,19,20]. We found that about half of the survival difference between Japanese and Whites was due to earlier stage at diagnosis among Japanese, the other half due to the greater proportion of Japanese living in Hawaii and the increased survival in Hawaii seen in some racial/ethnic groups. Choe et al found, in a survival analysis of national SEER data (with imputed nativity data) that US-born Asians experienced more favorable survival than foreign-born Asians [17]; as only 28% of Japanese (in California) are foreign-born [78] and they tend to be the most acculturated of other Asian subgroups, future research might focus on specific factors associated with being US-born Asian, or Japanese in particular, that confer a survival advantage. In a previous paper, we noted decreased stage-adjusted survival among Filipino males [19]; evidently, the poor survival was limited to both earlier years of diagnosis and younger Filipino men, thus, this pattern was not seen in this analysis.

Our race/ethnicity-specific survival models also revealed differential effects of some of the covariates on colorectal-specific and all-cause survival within each racial/ethnic group. Surprisingly, the area-based SES variables did not appear to be associated with colorectal cancer nor all-cause survival in most racial/ethnic groups. Only the measures poverty and education demonstrated marginally significant effects on survival among Whites, and income among Filipinos. The differential effect of marital status is also interesting: whereas single patients tended to have decreased survival than those who were married, this was not true for Blacks nor Filipinos. The quality of marital status information in the cancer registry is uncertain, so these results should be cautiously interpreted, but the beneficial effects of being married on cancer survival has been previously explored and is hypothesized to be due to greater spousal social support [79]. We also observed significantly decreased all-cause survival among males compared to females in nearly all racial/ethnic groups. As this gender difference was not seen for colorectal-specific survival among most racial/ethnic groups, this finding suggests that, relative to females, male colorectal patients experienced decreased survival due to causes of death other than colorectal cancer.

There were also differences in the effects of tumor characteristics and treatment. For example, histologic grade, reflecting tumor aggressiveness, did not appear to impact survival among Japanese and Filipinos. Although having any extent of surgical resection was uniformly associated with increased survival across all groups, a beneficial effect of radiation was only seen among Blacks. Chemotherapy was associated with increased survival in all groups, but not in Asians. These racial/ethnic differences in treatment impact on survival may reflect differential distributions of stage and colorectal cancer subsite among race/ethnic groups [63,75], or of a differential selection by physicians of healthier patients to receive these treatments [71,80]. In addition, because our measures of surgery, radiation, and chemotherapy are crude, the differences may also reflect differences in the quality of treatment. Further study examining survival patterns within specific stage and subsite, and incorporating more specific treatment data would be helpful. Overall, the results from these race/ethnicity-specific analyses support the value of evaluating each group on its own, as this type of analysis may reveal patterns that can result in more targeted and meaningful prevention efforts and strategies for improving survival for each group.

Stage remains the single most independently predictive factor on survival following diagnosis of colorectal cancer across and within racial/ethnic groups. For persons aged 50 or older, the American Cancer Society recommends one of five screening schedules, depending on the modality (fecal occult blood test (FOBT) yearly, flexible sigmoidoscopy or barium enema every 5 years, or colonoscopy every 10 years) [81]; however, data based on the California Health Interview Survey showed that only 53% of respondents reported having had an FOBT in the past year or sigmoidoscopy/colonoscopy in the past 5 years [82]. Moreover, Latinos reported lower screening for colorectal cancer (37%) compared to 56.4% among Whites [82]. Research on promoters and barriers to cancer screening consistently show that, among Asians and Latinos, screening is lower among recent migrants with limited English proficiency [82-87]. There is ongoing research to examine strategies to increase screening in different cultures [88]. Since early detection could potentially reduce some of these differences in survival, particularly among Hispanic men, it is clearly important to learn about barriers to screening among specific communities and to elucidate the most effective strategies for promoting screening in these communities.

Although our study provided a comprehensive evaluation of multiple factors in relation to survival across and within race/ethnic groups, our results may be limited by the quality of some of the SEER and Medicare data. For example, research of race/ethnic misclassification in medical admissions and registry data shows that although there is high consistency between self-report and hospital data for Whites and Blacks, consistency is lower for other groups, in particular Hispanics, American Indians, and certain Asian subgroups [25,33,89]. Our study may also be compromised by completeness of surgery, radiation, and, in particular, chemotherapy data. Comparisons of surgery and radiation recorded in SEER with other sources (Medicare admissions, physician survey) show that registry data are, for the most part, complete and do not vary greatly with regards to race/ethnicity [60,61,90-92]. Warren et al showed that physician claims data capture about 70% of the chemotherapy administered among colorectal cancer patients [62]; however, the prevalence of receipt of chemotherapy and of guideline treatment are lower than those seen in other studies [64,65,76] raising concerns about some under-ascertainment of chemotherapy in our data. Thus, we cannot discount the idea that our results, particularly the Black-White differences in survival, may be affected by completeness in our treatment data, particularly if it is differential across racial/ethnic groups. Our analyses could benefit from the addition of potentially useful information on insurance, hospital and physicians characteristics to investigate institutional factors that create unequal access to medical care [8,9,77,93,94]. Additionally, although information on colorectal cancer screening is theoretically available from Medicare claims data, insurance coverage for these screening modalities went into effect only recently [95,96] and the completeness may be further compromised by how providers bill for these procedures. Furthermore, we included patients diagnosed with colorectal cancer during the years 1992–1997 to allow for observation of a reasonable amount of time between diagnosis and follow-up; however, racial/ethnic survival patterns, particularly between Blacks and Whites, may have changed during this time. Data from the SEER registries showed that while 5-year survival improved among White males and females and Black females between 1992–1997 and 1997–2002, it has worsened slightly among Black males. Thus the survival disparity between Black and White males has increased [14], making the implications of our analysis even more noteworthy.

## Conclusion

Despite the inherent limitations in the data source used in the analyses, our study has strengths in being a comprehensive analysis of colorectal cancer survival – both in the enhanced inclusion of specific racial/ethnic groups and prognostic factors, gained from linkage to Medicare and Census data. We found evidence that, in the older Medicare population, racial/ethnic disparities in colorectal cancer survival may be reduced by increasing access to screening. However, among Blacks, survival disparities compared to Whites and other racial/ethnic groups persist even after accounting for a comprehensive set of sociodemographic, clinical, and treatment characteristics. Williams has proposed that the persistently worse health outcomes among Blacks may be attributable to broader societal discrimination in neighborhoods (i.e., residential segregation) and in health care delivery (i.e., institutional discrimination) [97,98]. With regards to colorectal cancer survival, additional research in areas that have received less attention, such as structural and institutional barriers [75,77], and of factors that have been proposed recently as having significant impacts on survival, including physical activity [99,100] and vitamin D [101-103], is necessary to identify the factors and mechanisms leading to the poorer outcomes among US Blacks. Population-based cancer registry data continue to be an invaluable resource for identifying and addressing racial/ethnic health disparities, however, expansion of the data through collection of additional data items and/or linkage to other data sources [104] is necessary for looking beyond traditional explanations, particularly if we hope to be able to reduce disparities in cancer outcomes.

## Competing interests

The author(s) declare that they have no competing interests.

## Authors' contributions

SLG designed the study, obtained the data, provided oversight of the data analyses, and drafted the manuscript. COM participated in the design of the study, the interpretation of the results, and helped to draft the manuscript. AS and SS participated in the design of the study and performed the statistical analyses. WS participated in the design of the study and helped to draft the manuscript. All authors read and approved the final manuscript.

## Pre-publication history

The pre-publication history for this paper can be accessed here:


